# TspanC8 tetraspanins differentially regulate the cleavage of ADAM10 substrates, Notch activation and ADAM10 membrane compartmentalization

**DOI:** 10.1007/s00018-015-2111-z

**Published:** 2015-12-19

**Authors:** Stéphanie Jouannet, Julien Saint-Pol, Laurent Fernandez, Viet Nguyen, Stéphanie Charrin, Claude Boucheix, Christel Brou, Pierre-Emmanuel Milhiet, Eric Rubinstein

**Affiliations:** Inserm, U935, 94807 Villejuif, France; Université Paris-Sud, Institut André Lwoff, 94807 Villejuif, France; Inserm, U1054, 34090 Montpellier, France; Université de Montpellier, CNRS, UMR5048, Centre de Biochimie Structurale, Montpellier, France; Institut Pasteur, Laboratoire “Signalisation et Pathogenèse”, 75015 Paris, France

**Keywords:** Membrane compartmentalization, Notch, ADAM10, Tetraspanin, Ectodomain shedding, Microdomain

## Abstract

The metalloprotease ADAM10 mediates the shedding of the ectodomain of various cell membrane proteins, including APP, the precursor of the amyloid peptide Aβ, and Notch receptors following ligand binding. ADAM10 associates with the members of an evolutionary conserved subgroup of tetraspanins, referred to as TspanC8, which regulate its exit from the endoplasmic reticulum. Here we show that 4 of these TspanC8 (Tspan5, Tspan14, Tspan15 and Tspan33) which positively regulate ADAM10 surface expression levels differentially impact ADAM10-dependent Notch activation and the cleavage of several ADAM10 substrates, including APP, N-cadherin and CD44. Sucrose gradient fractionation, single molecule tracking and quantitative mass-spectrometry analysis of the repertoire of molecules co-immunoprecipitated with Tspan5, Tspan15 and ADAM10 show that these two tetraspanins differentially regulate ADAM10 membrane compartmentalization. These data represent a unique example where several tetraspanins differentially regulate the function of a common partner protein through a distinct membrane compartmentalization.

**Electronic supplementary material:**

The online version of this article (doi:10.1007/s00018-015-2111-z) contains supplementary material, which is available to authorized users.

## Introduction

Members of the ADAM (a disintegrin and metalloprotease domain) family are membrane-anchored metalloproteases that mediate a proteolytic cleavage of various transmembrane proteins within their extracellular region. This process, referred to as ectodomain shedding, plays an important role in various cell and developmental processes [1, 2]. ADAM10 mediates the ectodomain shedding of more than 40 transmembrane proteins, including cytokine and growth factor precursors, as well as adhesion proteins such as E and N-cadherins [2]. ADAM10-mediated cleavage of the amyloid precursor protein (APP) prevents the formation of the amyloid peptide Aβ, a major component of amyloid plaques observed in Alzheimer’s disease [3]. ADAM10 is also the main protease for the cleavage of Notch receptors at a site called S2 following ligand binding. This step is a prerequisite for a second cleavage at the S3 site by the γ-secretase complex that results in the release of Notch intracellular domain (NICD), which translocates to the nucleus and regulates the transcription of Notch target genes [4–7]. Importantly, ADAM10-deficient mice die during the development, and its tissue-specific ablation yields abnormalities in various organs that are associated with a defect in Notch signaling [8–11].

Ectodomain shedding is a tightly regulated process and has been shown to be stimulated by various stimuli. For example, PKC activators and various GPCR ligands have been shown to strongly stimulate ADAM17-dependent shedding of various substrates, including growth factors and cytokines [1, 12]. The activity of ADAM10 towards various substrates has been shown to be stimulated by calcium ionophores, activation of P2X7 receptor as well as by mAb directed against several members of the tetraspanin superfamily [12–14]. The differential ability of ADAM10 and ADAM17 to support the proteolysis and activation of normal and mutant forms of Notch also suggests a tight regulation of these proteases. Indeed, ADAM10 is essential and ADAM17 dispensable for ligand-dependent Notch activation in cellular models [5–7]. This is consistent with the lack of Notch-loss of function phenotype in ADAM17-null animals [15]. In contrast, mutant forms of Notch that are active independently from the presence of ligand can be cleaved at the S2 site by other proteases including ADAM17 [5, 6].

The expression of ADAM10 and Notch activity are regulated by several members of the tetraspanin superfamily [16–19]. Tetraspanins are expressed by all metazoans, and are characterized by four transmembrane domains that flank two extracellular domains of unequal size, conserved key residues, and a specific fold of the large extracellular domain. Genetic studies in humans or mice have shown their key role in a number of physiological processes including reproduction, vision, immunity, kidney function, muscle regeneration and mental capacity [20–22]. A major feature of these molecules is to associate with many other integral proteins, thus organizing a dynamic network of interactions referred to as the “tetraspanin web” or tetraspanin-enriched microdomains [20–22]. The organization of this web has been resolved, at least in part, with tetraspanins interacting directly with a limited number of partner proteins to form primary complexes which in turn associate with one another. Several tetraspanin/partner pairs have been identified. For example, CD151 associates directly with the laminin-binding integrins α3β1 and α6β1 [23, 24], and CD9 and CD81 share two common partners, CD9P-1 and EWI-2, two related Ig domain proteins [25–28]. Tetraspanins regulate various properties of the molecules they associate with, including their trafficking, the binding of ligands, downstream signaling, and for ectoenzymes, their enzymatic activity [20–22, 29].

We and others have recently demonstrated that ADAM10 has six tetraspanin partners, which mediate its exit from the ER and belong to a subgroup of tetraspanins having eight cysteines in the largest of the two extracellular domains and referred to as TspanC8 [16, 18, 19]. This level of regulation appears to be important for Notch signaling and is evolutionary conserved. Indeed, silencing Tspan5 and Tspan14 reduced Notch activity in a human cell line, in association with a reduction of ADAM10 surface expression [16]. Mutations of the TspanC8 tetraspanin Tsp-12 in *Caenorhabditis elegans* genetically interacted with Notch or ADAM10 mutations [17]. Finally, depletion of the three *Drosophila* TspanC8 tetraspanins impaired several Notch-dependent developmental processes, Notch activity and ADAM10 subcellular localization in vivo [16].

Direct association of ADAM10 with several tetraspanin partners suggests that some of its properties could be regulated differently depending on the tetraspanin with which it is associated. We show here that the TspanC8 tetraspanins Tspan5, Tspan14, Tspan15 and Tspan33 have a different impact on ADAM10-dependent functions. In particular, Tspan33 and Tspan15 appear to be negative regulators of ligand-induced Notch activity. We also show that Tspan5 or Tspan15 differentially affect the membrane compartmentalization of ADAM10 as shown by confocal microscopy analysis, single molecule tracking and the analysis of their repertoire of co-immunoprecipitated molecules. These data present strong evidence that tetraspanins can regulate the function of their partner proteins by acting on their membrane compartmentalization.

## Results

### Tspan15 is a negative regulator of Notch activity

**Fig. 1:**
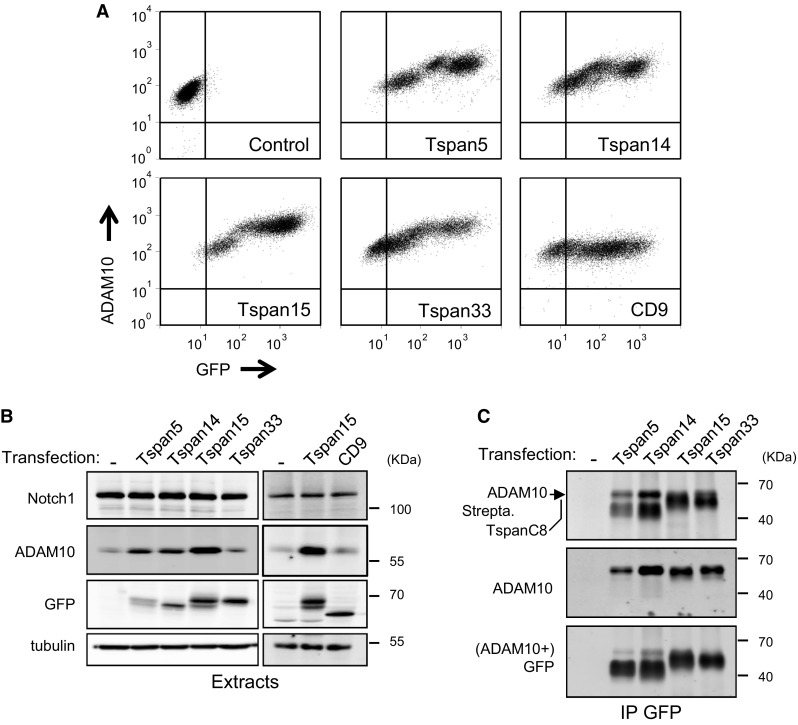
Expression of four TspanC8 tetraspanins in U2OS-N1 cells. **a** Flow-cytometry analysis of the surface expression of ADAM10 in U2OS-N1 cells stably expressing GFP-tagged TspanC8 tetraspanins or CD9. **b** Western-blot analysis of the expression of Notch1, ADAM10 and tetraspanins in U2OS-N1 cells stably expressing GFP-tagged TspanC8 or CD9. **c** After biotin labeling of surface proteins, U2OS-N1 cells stably expressing or not GFP-tagged Tspan5, Tspan14, Tspan15 and Tspan33 were lysed and the interaction of these tetraspanins with ADAM10 was analyzed by co-immunoprecipitation using GFP-trap beads and Western blot. The major 68 kDa band revealed by the anti-ADAM10 mAb perfectly overlapped with the band labeled ADAM10 in the upper panel (*black arrowhead*). Immunoblotting with the GFP antibody was done after ADAM10 immunoblotting. All experiments were performed at least three times with similar outcome

**Fig. 2:**
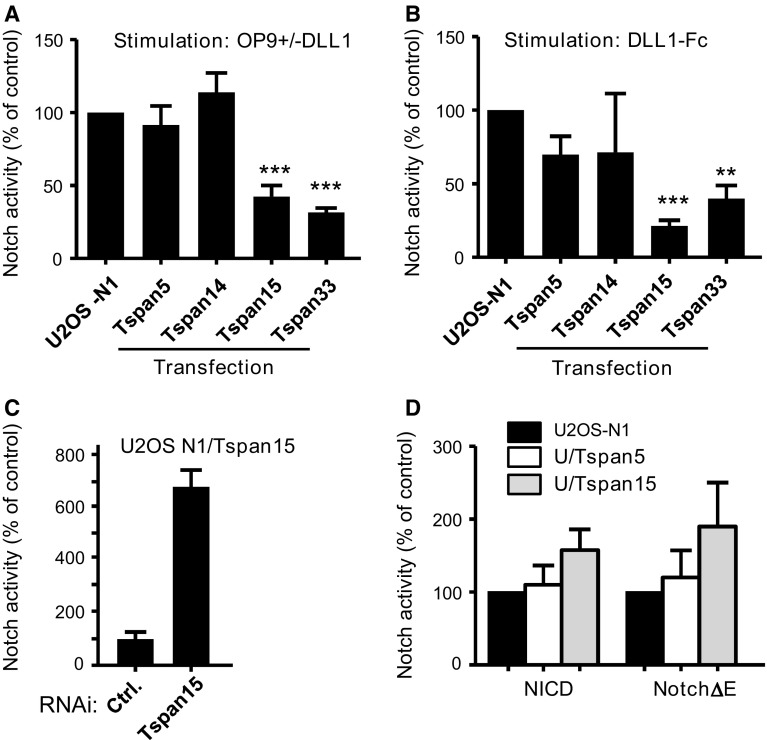
Transfection of Tspan15 and Tspan33 reduces Notch activity in U2OS-N1 cells. **a** Notch activity, measured using a CSL reporter luciferase assay, of U2OS-N1 cells stably expressing or not Tspan5, Tspan14, Tspan15 or Tspan33. Notch was activated by incubation of the cells with OP9-DLL1 cells for 20–24 h. The *graph* shows the mean ± SEM of ten (Tspan5, Tspan15) or four (Tspan14, Tspan33) independent experiments in duplicate. In each experiment, the signal obtained is expressed as a percentage of the signal observed for non-transfected U2OS cells. **b** Notch activity of U2OS-N1 cells stably expressing or not Tspan5, Tspan14, Tspan15 or Tspan33. Notch was activated by incubation of the cells on DLL1-Fc-coated tissue culture plates for 20–24 h. The* graph* shows the mean ± SEM of five (Tspan5, Tspan15), four (Tspan33) or two (Tspan14) independent experiments in duplicate. Each experiment is normalized on the signal obtained for non-transfected U2OS cells. **c** U2OS-N1/Tspan15 were treated with a control siRNA or a siRNA targeting Tspan15 before analysis of Notch activity. The *graph* shows the mean ± SEM of three independent experiments performed in duplicate. **d** U2OS-N1 cells stably expressing or not Tspan5 or Tspan15 were transiently transfected with NICD and Notch1-ΔE constructs. Notch activity was determined using the luciferase assay. The* graph* shows the mean ± SEM of two independent experiments performed in duplicate. ***p < 0.001 and **p < 0.01 as compared with control cells

We have previously demonstrated that silencing Tspan5 and Tspan14 in U2OS cells transduced with human Notch1 (U2OS-N1) decreased ADAM10 surface expression levels and Notch activity. We could not test the role of Tspan15 and Tspan33 in these cells which do not express these two tetraspanins. To directly compare the effect of Tspan5, Tspan14, Tspan15 and Tspan33 on Notch activity, we stably expressed these TspanC8 in U2OS-N1 cells. All 4 tetraspanins were expressed at the cell surface as determined by labeling with membrane impermeable biotin (Fig. 1), associated with endogenous ADAM10 and stimulated a 3- to 5-fold increase in ADAM10 surface expression levels. In contrast, there was no change of Notch expression (Fig. 1). To examine the impact of the expression of these TspanC8 on ligand-induced Notch activity, the different cell lines were co-cultured with OP9 cells expressing or not the Notch ligand DLL1. The expression of Tspan5 or Tspan14 had no significant effect on Notch activity. In contrast, U2OS-N1 cells expressing Tspan15 or Tspan33 showed a ~60 % decrease in OP9-DLL1-induced Notch activity as compared to U2OS-N1 cells (Fig. 2a). In addition, cells transfected with Tspan15 and Tspan33 also showed diminished Notch signaling in response to immobilized DLL1, indicating that these tetraspanins do not modulate Notch signaling by changing the interaction of U2OS-N1 cells with OP9-DLL1 cells (Fig. 2b). In addition, the transfection of Tspan15 or Tspan33 did not change the expression level of endogenous Tspan5 and Tspan14, as determined by RT-qPCR (data not shown). Additional experiments were performed to further characterize the effect of Tspan15 on Notch signaling. The inhibition of Notch signaling is not due to the selection of a sub-population of U2OS-N1 cells having a lower ability to respond to Notch activation because a second independent cell population of cells expressing Tspan15 showed similar decrease in Notch signaling (Fig. S1). In addition, silencing Tspan15 in U2OS-N1/Tspan15 cells restored Notch signaling (Fig. 2c). Tspan15 expression did not reduce the activity of two constitutively active Notch constructs (Fig. 2d): NICD, which corresponds to the intracellular domain of Notch1 lacking the PEST domain, and Notch1-ΔE, which contains a short extracellular stub, the transmembrane domain and the intracellular domain of Notch1 without the PEST domain [30–32]. The activity of both constructs is independent from ADAM10 activity, whereas the activity of Notch1-ΔE, but not NICD, requires γ-secretase activity. Thus, Tspan15 acts at a pre-γ-secretase step.

**Fig. 3:**
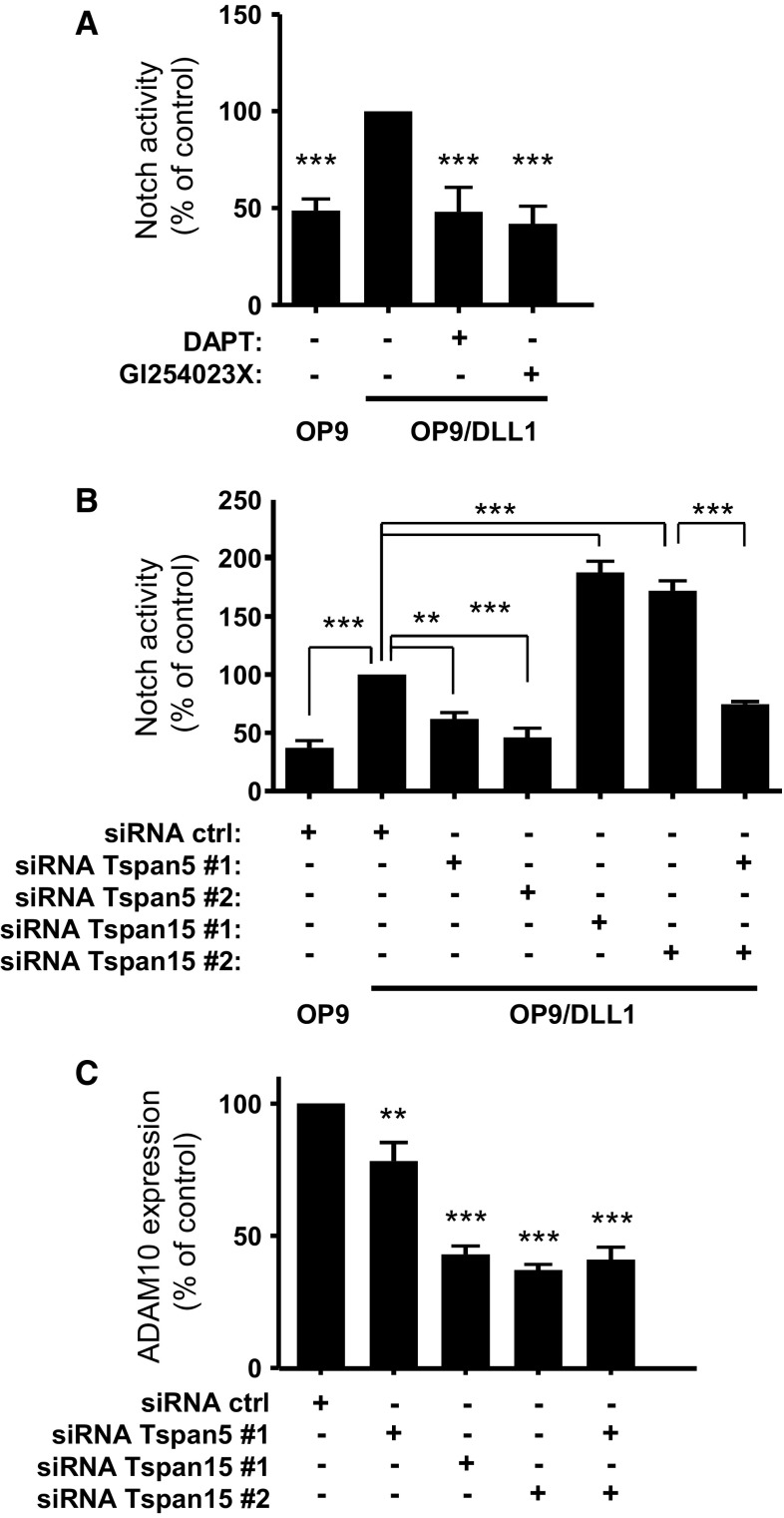
Tspan5 and Tspan15 are positive and negative regulators of Notch activity in PC3 cells. **a** Notch activity in PC3 cells measured using a CSL reporter luciferase assay. PC3 cells transfected with the reporter construct were incubated with OP9 or OP9-DLL1 cells for 20–24 h. The cells were incubated or not with the ADAM10 inhibitor GI254023X (3 µM) or the γ-secretase inhibitor DAPT (3 µM). The *graph* shows the mean ± SEM of three independent experiments in duplicate. **b** Notch activity, measured using a CSL reporter luciferase assay, of PC3 cells treated with the indicated siRNA. Notch was activated by incubation with OP9-DLL1 cells, or with OP9 cells as a control for endogenous activity. The *graph* shows the mean ± SEM of three independent experiments performed in duplicate. **c** Flow-cytometric analysis of the surface expression of ADAM10 in PC3 cells treated with the indicated siRNA. The* graph* shows the mean ± SEM of four independent experiments. ***p < 0.001 and **p < 0.01 as compared with control cells or other conditions

We then investigated whether endogenous Tspan15 has also an inhibitory effect on Notch signaling. PC3 prostate cancer cells have been shown to express Notch-1 at the protein level [33]. RT-qPCR analysis has shown that these cells express mainly Tspan15 and Tspan5 but no Tspan14 [16]. As shown in Fig. 3a, Notch activity, determined using the luciferase assay, was 2.5 times higher in cells co-cultured with OP9-DLL1 cells than in cells co-cultured with OP9 cells. This activation is due to canonical Notch activation because it was blocked by DAPT, a γ-secretase inhibitor, and by the ADAM10 inhibitor GI254023X (Fig. 3a). Silencing Tspan15 in these cells, with two previously validated siRNA, decreased Tspan15 mRNA levels by ~90 % [16], decreased ADAM10 surface expression levels by ~60 % (Fig. 3c), but increased Notch activity induced by OP9-DLL1 cells twofold (Fig. 3b). In contrast, silencing Tspan5 had less impact on ADAM10 expression (~20 % reduction in surface expression levels) but reduced Notch activity in response to OP9-DLL1 cells by ~50 % (Fig. 3b). Tspan5 depletion also prevented the increase in Notch signaling observed upon silencing Tspan15.

Altogether these data indicate that the different TspanC8 have a different impact on Notch signaling. In particular, Tspan15 is a negative regulator of this signaling, acting at a step upstream the γ-secretase step, probably by regulating ADAM10 activity.

### TspanC8 tetraspanins differentially regulate the cleavage of ADAM10 substrates

**Fig. 4:**
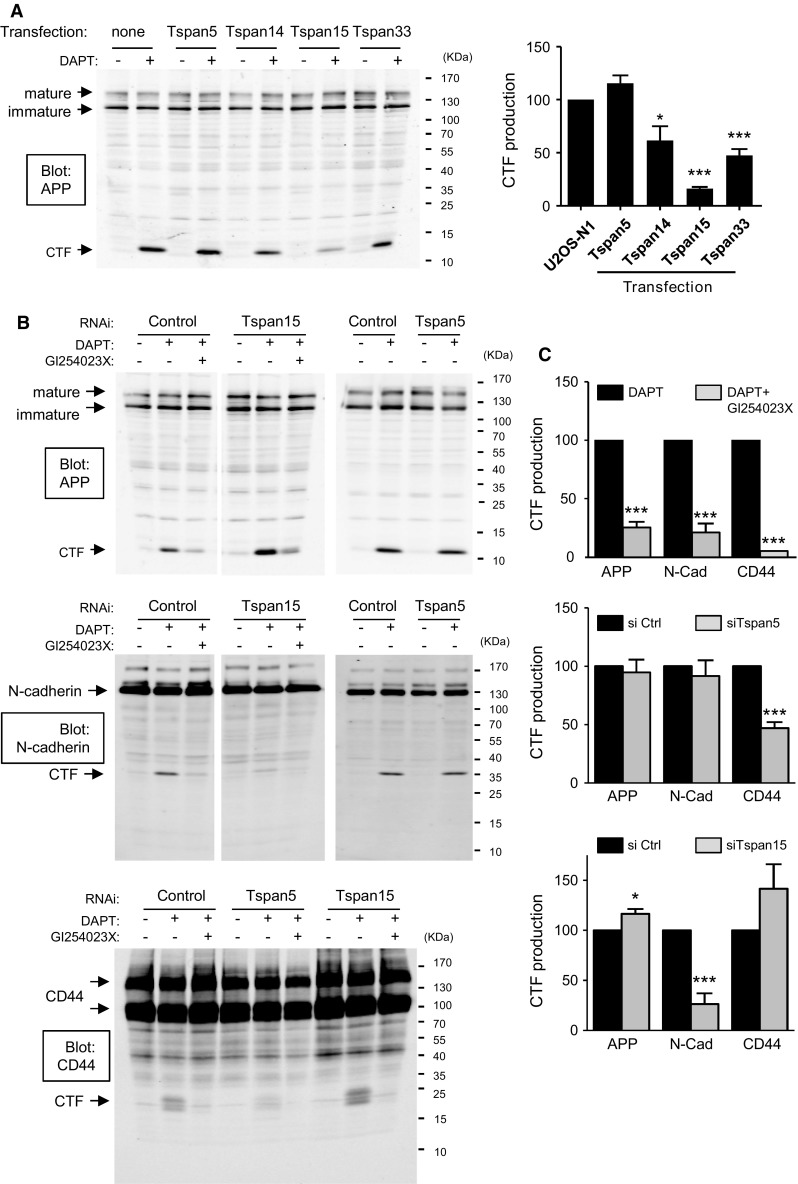
TspanC8 differentially regulate the cleavage of ADAM10 substrates. **a** Western-blot analysis of APP in U2OS cells expressing or not the various TspanC8, after incubation for 24 h in DMSO or the γ-secretase inhibitor DAPT. The *graph on the right* shows a quantification of the production of APP CTF (mean ± SEM) of three independent experiments. **b** Representative Western-blot analysis of APP (*top*), N-cadherin (*middle*) and CD44 (*bottom*) in PC3 cells treated with a control siRNA or siRNA targeting Tspan5 and Tspan15, and incubated for 24 h with DMSO, DAPT or a combination of DAPT and the ADAM10 inhibitor GI254023X. **c** Quantification (mean ± SEM) of the effect of GI254023X, and siRNA targeting Tspan5 or Tspan15 on the production of APP, N-cadherin (N-cad) and CD44 CTF. The experiments were performed twice with three different siRNA for each tetraspanin. To take into consideration the potential variability of the effect of different siRNA, the mean and SEM were calculated on the data obtained for all three siRNA

We then tested whether the expression of the different TspanC8 in U2OS cells modified the cleavage of other well-characterized ADAM10 substrates. For this purpose we analyzed the production of the carboxy-terminal membrane stub (CTF) generated upon cleavage using antibodies to the cytoplasmic domain. To prevent degradation of this fragment, cells were treated with DAPT, a γ-secretase inhibitor. As shown in Fig. 4a, U2OS-N1 cells expressing Tspan15 showed a 80 % reduction in APP CTF production as compared to parental cells. A partial reduction of APP CTF production was also observed for cells expressing Tspan14 or Tspan33, but not for cells expressing Tspan5.

To examine the role of endogenous Tspan5 and Tspan15, we studied the cleavage of ADAM10 substrates in PC3 cells (Fig. 4b, c). In these cells, the production of APP, N-cadherin and CD44 CTF were dependent on ADAM10 activity as shown by the inhibition by the ADAM10 inhibitor GI254023X (Fig. 4b, c, top). Tspan15 silencing by three different siRNA reduced the production of N-cadherin CTF by ~75 % on average, and slightly increased the production of APP CTF (Fig. 4b, c, bottom). We were unable to conclude about the role of Tspan5 in regulating the cleavage of APP or N-cadherin in this model because the three different siRNA used had a different effect, yielding no change on average. (Fig. 4b, c, middle). In contrast, all three Tspan5 siRNA reduced the production of CD44 CTF (Fig. 4b, c, middle; on average the reduction of CD44 CTF production is ~55 %). The production of CD44 CTF was not modified upon Tspan15 silencing (Fig. 4b, c, bottom).

Altogether, these data indicate that the different TspanC8 tetraspanins have a different impact on the cleavage of several ADAM10 substrates.

### Differential membrane compartmentalization of ADAM10 according to the expression of Tspan5 or Tspan15

**Fig. 5:**
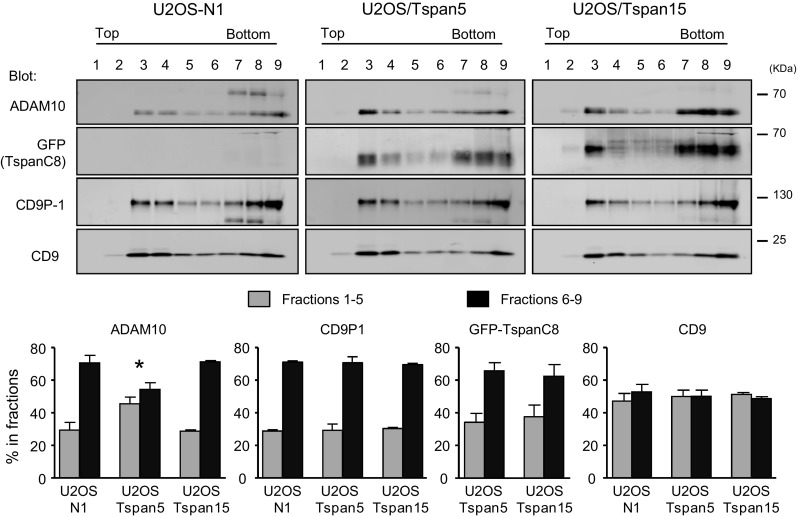
Tspan5 and Tspan15 differentially affect ADAM10 solubilization. U2OS cells transfected with Tspan5 or Tspan15 were lysed in the presence of Brij98. The lysates were directly subjected to equilibrium density gradient centrifugation. Gradient fractions were collected and analyzed by Western blot as indicated. The* graphs* show the relative abundance (mean ± SEM) of the indicated proteins in low (fractions *1*–*5*) and high (fractions *6*–*9*) density fractions in three independent experiments. *p < 0.05 as compared with the other cell types

We reasoned that the differential activity of TspanC8 on Notch activity could be due to a different compartmentalization of ADAM10. Sucrose gradient fractionation was used to determine the impact of these proteins on the membrane environment of ADAM10. In initial experiments, we found that ADAM10 (and the other molecules tested here) was nearly completely solubilized after lysis using Brij97 (data not shown). In contrast, a fraction of ADAM10 partitioned into the light fractions of sucrose gradients after lysis with a milder detergent, Brij98 (Fig. 5). Importantly, the fraction of ADAM10 present in low density fractions was higher in cells transfected with Tspan5 than in cells transfected with Tspan15 or in parental U2OS-N1 cells, indicating a change in the membrane environment of ADAM10 in these cells. As a control, the partitioning into the different fractions of CD9 and CD9P-1, a CD9 and CD81 molecular partner, was similar in the different cell lines.

**Fig. 6:**
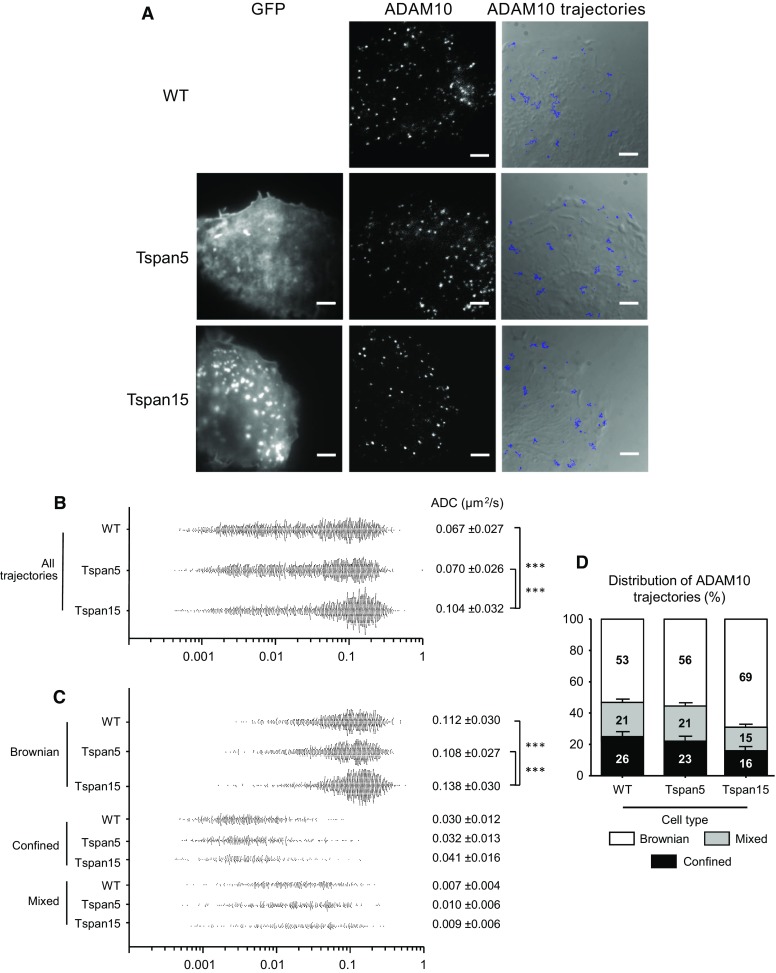
Tspan5 and Tspan15 differentially affect ADAM10 dynamic behavior. **a** TIRF microscopy analysis of U2OS-N1 cells expressing or not GFP-tagged Tspan5 or Tspan15. *Left column* GFP signal; *middle* labeling of ADAM10, using a concentration of anti-ADAM10 Fab allowing single molecule detection. The images shown here are the first frame of the movies shown as supplementary information. *Right column* superimposition of 30 randomly selected ADAM10 trajectories with the DIC image of cells acquired before tracking. *Bar* 5 µm. **b** Distribution of the apparent diffusion coefficients (ADC) calculated for all individual ADAM10 molecules analyzed in U2OS-N1 cells expressing or not Tspan5 or Tspan15. Each *dot* represents one trajectory and 1500 trajectories are shown for each cell type. The mean ADC value ± SEM are indicated on the *right*. *Triple asterisks* indicate that the difference between the two cell types are significant with a *p* value below 0.001 as determined by the Mann–Whitney *U* test. **c** Distribution of the apparent diffusion coefficients (ADC) calculated for individual ADAM10 molecules according to their type of motion (Brownian, confined, mixed). *Triple asterisks* difference between the two cell types are significant with a *p* value below 0.001 as determined by the Mann–Whitney *U* test. **d** Histogram representing the percentage of ADAM10 molecules undergoing Brownian, confined and mixed trajectories relative to the total number of trajectories, in U2OS-N1 cells expressing or not Tspan5 or Tspan15

Dynamics and partitioning of ADAM10 in U2OS-N1 cells expressing or not Tspan5 or Tspan15 were next investigated using single-molecule tracking (SMT). In this technique, the labeling of a low number of molecules allows individual molecules to be optically isolated and their position accurately determined. Frame by frame positioning of the proteins allows reconstruction of their trajectories, calculation of their apparent diffusion coefficient (ADC) as well as the determination of their mode of diffusion. Similar to other membrane proteins, including CD9 and CD81 [34–36], the distribution of ADAM10 ADC was large (Fig. 6a) and three modes of diffusion were identified (Fig. 6b, c): (1) pure Brownian diffusion, (2) pure confined or restricted diffusion and (3) diffusion with different combinations of Brownian and confined modes referred to as “mixed trajectories”. The behavior of ADAM10 molecules in cells expressing Tspan15 was different from that of parental cells or cells expressing Tspan5. The average ADC of ADAM10 was significantly higher in cells expressing Tspan15 than in parental cells or cells expressing Tspan5 [0.104 ± 0.032 µm^2^/s versus 0.067 ± 0.027 (U2OS-N1) and 0.070 ± 0.026 (Tspan5) (mean ± SEM, *n* = 1500, *p* < 0.0001)] (Fig. 6b). This is due to a higher ADC of molecules displaying a Brownian motion [0.138 ± 0.030 versus 0.112 ± 0.030 µm^2^/s (U2OS-N1) and 0.108 ± 0.027 (Tspan5), *n* = 1035, 795 and 840, *p* < 0.0001] as well as to a lower percentage of molecules undergoing confined and mixed trajectories [16/15 versus 26/21 % (U2OS-N1) and 23/21 % (Tspan5), Fig. 6d], the diffusion of which is slower (Fig. 6c). Altogether, these data show that the membrane environment of ADAM10 was modified upon Tspan15 expression, and indicate that the membrane compartmentalization of ADAM10 is differentially regulated by Tspan5 and Tspan15.

### The repertoires of proteins co-immunoprecipitated with Tspan5 and Tspan15 display quantitative and qualitative differences

The above data suggest that Tspan5 and Tspan15 target ADAM10 to different membrane compartments. As a first step to identify these compartments, we compared by label-free quantitative mass-spectrometry the repertoire of plasma membrane-resident integral proteins co-immunoprecipitated with Tspan5, Tspan15 or CD9 (a classical tetraspanin) after Brij97 lysis, a detergent that preserves any number of interactions inside the tetraspanin web, including tetraspanin to tetraspanin interactions. Most previous analyses of tetraspanin-associated proteins were performed after immunoprecipitation using anti-tetraspanin antibodies. In the absence of good antibodies to Tspan5 or Tspan15, we had to rely on an alternative approach. To evaluate the suitability of GFP-trap pull down of GFP-tagged tetraspanins, we compared the pattern of cell membrane proteins co-immunoprecipitated with CD9 (from U2OS-N1 cells) using a CD9 mAb, or with CD9-GFP (from U2OS N1 cells stably expressing CD9-GFP) using GFP-trap beads. These two approaches yielded very similar sets of co- immunoprecipitated proteins (supplementary Table 1), indicating the appropriateness of the GFP-trap approach. It should be, however, pointed out that several tetraspanins (CD81 or CD82 for example) were not or less well detected using the GFP-trap approach. Of note, both Tspan14 and Tspan5 were detected in the CD9 immunoprecipitation performed with the CD9 mAb (supplementary Table 1).

**Fig. 7:**
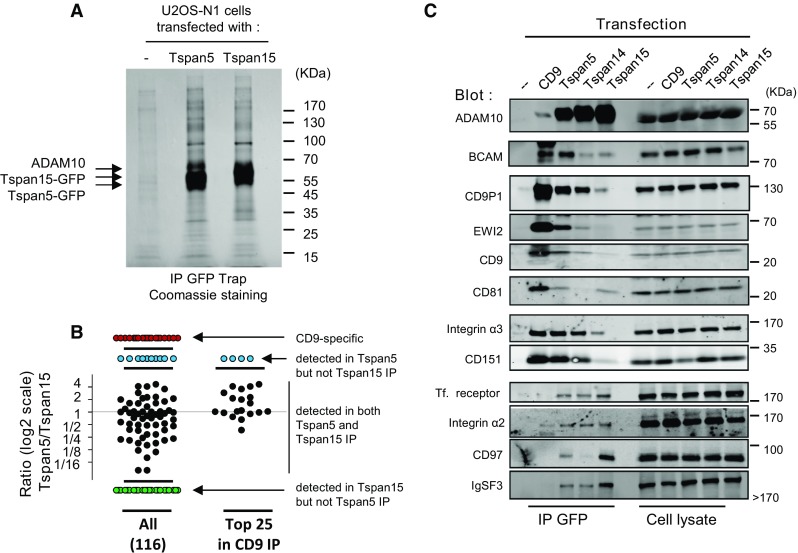
Analysis of the repertoire of proteins co-immunoprecipitated with Tspan5, Tspan15 or CD9. **a** The proteins co-immunoprecipitated with Tspan5 or Tspan15 from Brij97 lysates of U2OS-N1/Tspan5 or U2OS-N1/Tspan15 using GFP Trap beads were separated by SDS/PAGE and visualized by Coomassie blue staining. As a control, the same procedure was applied to parental U2OS cells. **b** Distribution of the different integral plasma membrane proteins identified in CD9, Tspan5 or Tspan15 immunoprecipitates by quantitative mass-spectrometry, according to their relative abundance in the Tspan5 and Tspan15 immunoprecipitates. Each *dot* corresponds to a protein. Those proteins that are present in both immunoprecipitates are represented according to their ratio in the Tspan5 IP versus the Tspan15 IP. The proteins immunoprecipitated with Tspan5 but not Tspan15, with Tspan15 but not Tspan5, and proteins only found in the CD9 IP are also shown. The *left part* of the graph shows all 116 identified proteins. The *right part* shows the relative levels in the Tspan5 and Tspan15 IP of the 25 most abundant proteins in the CD9 IP. Note that most of them are more efficiently co-immunoprecipitated with Tspan5 than with Tspan15. **c** U2OS-N1 cells transfected or not with the indicated tetraspanins were lysed in Brij 97 before immunoprecipitation using GFP Trap beads. The composition of the immunoprecipitates was analyzed by western-blot

Table 1 shows the most abundant integral proteins present at the plasma membrane co-immunoprecipitated with CD9, Tspan5 or Tspan15 using the GFP-trap approach. Importantly, ADAM10 was by far the most abundant protein co-immunoprecipitated with Tspan5 and Tspan15, indicating that it is the major protein associating with these tetraspanins in U2OS-N1 cells. Tspan15 co-immunoprecipitated at high levels a number of proteins that were not or poorly co-immunoprecipitated with the other two tetraspanins. In contrast, no proteins were specifically co-immunoprecipitated with Tspan5. Importantly, a number of proteins known to associate (directly or indirectly) with CD9 were better co-immunoprecipitated with Tspan5 than with Tspan15 (Table 1; supplementary Table 1; Fig. 7b). These proteins include the CD9 and CD81 partners CD9P-1/EWI-F and EWI-2, as well as the two subunits of the integrin α3β1, a CD151 partner. Western-blot analysis of the immunoprecipitates confirmed that these molecules are better immunoprecipitated with Tspan5 than with Tspan15 (Fig. 7c). This is consistent with the better immunoprecipitation of CD9 and CD151 with Tspan5 than with Tspan15. Tspan14 co-immunoprecipitated intermediate levels of these proteins. Among the proteins that were found to better co-immunoprecipitate with Tspan15, we validated the interaction with CD97 and IgSF3 by Western blot (Fig. 7c). The interaction with other identified proteins, such as APMAP, could not be validated because we did not find antibodies suitable for Western-blot analysis. We also validated that Tspan5 and Tspan15 co-immunoprecipitated a similar level of the transferrin receptor and of the integrin α2β1. The proportion of these receptors co-immunoprecipitated with tetraspanins was, however, lower than that of the other proteins tested. Finally, among the ADAM10 substrates tested in this study, N-cadherin (cadherin-2) and CD44 were better co-immunoprecipitated with Tspan15 and Tspan5, respectively (supplementary Table 1).

**Table 1:** Major plasma membrane associated integral proteins co-immunoprecipitated with CD9, Tspan5 or Tspan15

Protein name	Gene name	Protein abundance (area × 10^6^) (number of unique peptides)					
		CD9		Tspan5		Tspan15	
CD9-specific							
**Sodium/potassium-transporting ATPase subunit beta-1**	ATP1B1	71.37	(3)	ND		ND	
**Monocarboxylate transporter 8 (MCT 8)**	SLC16A2	40.67	(2)	ND		ND	
Tsp5 ≈ Tsp15							
Disintegrin and metalloproteinase domain-containing protein 10	ADAM10	27.69	(5)	3795.00	(18)	4232.00	(31)
Transferrin receptor protein 1	TFRC	81.55	(23)	50.40	(16)	49.01	(19)
CD9 antigen	CD9	7170.00	(5)	49.59^a^	(1)	48.51^a^	(1)
4F2 cell-surface antigen heavy chain	SLC3A2	44.10	(16)	32.84	(9)	20.20	(6)
**Sodium/potassium-transporting ATPase subunit alpha-1**	ATP1A1	145.10	(25)	26.19	(15)	27.30	(13)
Disintegrin and metalloproteinase domain-containing protein 17	ADAM17	45.01	(8)	24.14	(5)	24.56	(4)
HLA class I histocompatibility antigen, B alpha chain	HLA-B	14.22	(2)	23.77	(1)	34.52	(2)
Integrin alpha-2	ITGA2	7.76	(2)	21.53	(15)	15.90	(6)
*Basal cell adhesion molecule*	*BCAM*	*22.46*	*(12)*	*18.76*	*(6)*	*18.25*	*(3)*
**Teneurin-3**	TENM3	61.92	(31)	18.54	(6)	24.03	(9)
Tetraspanin-14	TSPAN14	ND		4.42	(1)	5.09	(2)
**Sodium/potassium-transporting ATPase subunit beta-3**	ATP1B3	55.54	(3)	3.54^a^	(1)	5.33^a^	(1)
Tsp5 > Tsp15							
Tetraspanin-5	TSPAN5	3.89	(1)	3270.00	(6)	7.80^a^	(1)
Integrin beta-1	ITGB1	514.40	(16)	423.40	(17)	103.50	(9)
Integrin alpha-3	ITGA3	515.30	(23)	288.00	(17)	99.67	(14)
CD44 antigen	CD44	89.57	(5)	28.47	(2)	14.25	(3)
**Erythrocyte band 7 integral membrane protein**	STOM	9.02	(3)	28.43	(9)	2.90	(3)
Tetraspanin-9	TSPAN9	21.06	(2)	28.09	(2)	6.07	(2)
Prostaglandin F2 receptor negative regulator (CD9P-1/EWI-F)	PTGFRN	808.80	(28)	26.32	(8)	7.42^a^	(1)
CD151 antigen	CD151	12.10	(1)	26.09	(1)	ND	
Syntaxin-4	STX4	79.67	(6)	21.36	(6)	9.50^a^	(1)
CD166 antigen	ALCAM	10.17	(4)	21.33	(1)	10.09	(2)
Low-density lipoprotein receptor	LDLR	ND		21.02	(1)	9.15^a^	(3)
**Cell surface glycoprotein MUC18**	MCAM	4.52	(1)	20.11	(8)	ND	
**Latrophilin-2**	LPHN2	24.63	(12)	18.62	(2)	ND	
*Integrin alpha*-*6*	*ITGA6*	*114.70*	*(27)*	*17.17*	*(9)*	*15.82* ^a^	*(6)*
Immunoglobulin superfamily member 8 (EWI-2)	IGSF8	218.00	(4)	14.66	(5)	6.87^a^	(2)
**Cytoskeleton-associated protein 4**	CKAP4	ND		36.13	(11)	20.22	(4)
Tsp15 > Tsp5							
**Platelet-derived growth factor receptor beta**	PDGFRB	1.98	(3)	6.70	(1)	87.70	(12)
**CD97 antigen**	CD97	4.35	(2)	22.24	(5)	76.17	(9)
Matrix metalloproteinase-14 (MT1-MMP)	MMP14	16.05	(4)	7.51^a^	(1)	65.51	(3)
*Syntaxin*-*3*	*STX3*	*40.02*	*(4)*	*19.91*	*(6)*	*54.13*	*(3)*
**Teneurin-2**	TENM2	19.51	(17)	1.96	(1)	52.10	(2)
**Sn1-specific diacylglycerol lipase beta**	DAGLB	3.19	(2)	7.59	(1)	47.95	(6)
**Zinc transporter ZIP14**	SLC39A14	14.56	(2)	6.39	(1)	27.73	(2)
**Immunoglobulin superfamily member 3**	IGSF3	1.74	(1)	5.27	(3)	24.10	(15)
***Chondroitin sulfate proteoglycan 4***	*CSPG4*	*ND*		*5.07*	*(6)*	*43.96*	*(26)*
**Protein sidekick-2**	SDK2	ND		13.11	(4)	36.49	(5)
**C-type mannose receptor 2**	MRC2	ND		12.50	(6)	33.23	(8)
**Integral membrane protein 2C (Transmembrane protein BRI3)**	ITM2C	ND		10.37	(2)	21.05	(1)
Tetraspanin-15	TSPAN15	ND		ND		6946.00	(8)
**Adipocyte plasma membrane-associated protein**	APMAP	ND		ND^b^		274.40	(10)
**Discoidin, CUB and LCCL domain-containing protein 2**	DCBLD2	ND		ND		38.92	(3)
**Ephrin type-B receptor 2**	EPHB2	ND		ND		24.30	(8)
**Latrophilin-1**	LPHN1	ND		ND		21.46	(10)
**CD276 antigen**	CD276	29.80	(2)	ND^b^		22.90	(3)
**Dystroglycan**	DAG1	23.08	(6)	ND		11.27^a^	(2)
CD81 antigen	CD81	14.00	(1)	ND^b^		5.77	(1)

The table show the most abundant integral proteins known to be present at the plasma membrane and co-immunoprecipitated with GFP-tagged CD9, Tspan5 or Tspan15 using GFP trap beads. Only proteins identified in two experiments with an area ≥2 × 10^7^ in at least one of the IP are shown, except for tetraspanins. The values shown in this table are those obtained in the experiment performed using the highest number of cells. The proteins in italic correspond to proteins for which the relative ratio in the Tspan5 and Tspan15 IP are different in the two experiments. Proteins in bold character correspond to proteins not previously demonstrated to associate with tetraspanins. A complete list of proteins obtained in the two experiments is provided in supplementary table I

^a^Not detected in the second experiment in which less cells are used

^b^Detected in the second experiment

Altogether, these data indicate that the repertoire of integral membrane proteins co-immunoprecipitated with Tspan5 more closely resembles that of CD9 than the repertoire of Tspan15-co-immunoprecipitated proteins.

### Tspan5 and Tspan15 expressions have a different impact on the interaction of ADAM10 with other integral proteins

**Fig. 8:**
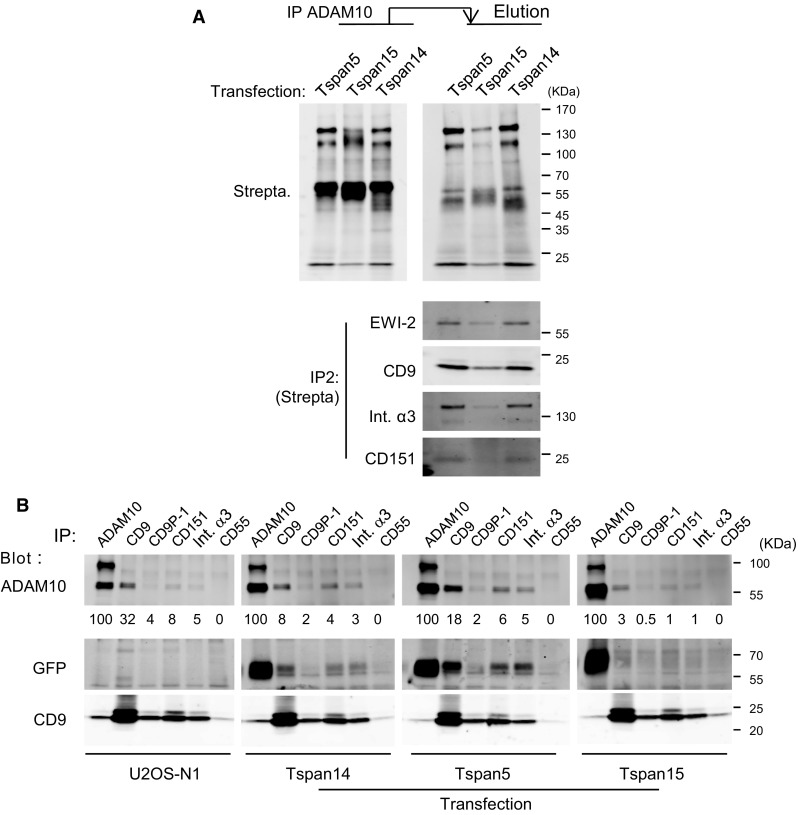
Expression of Tspan15 weakens the association of ADAM10 with classical components of the tetraspanin web. **a** After biotin labeling of surface proteins, U2OS-N1 cells expressing Tspan5, Tspan14 or Tspan15 were lysed in the presence of Brij 97 and ADAM10 was immunoprecipitated using mAb 11G2-coupled Sepharose beads. The co-immunoprecipitated proteins were eluted in RIPA buffer, and selected proteins were immunoprecipitated using specific antibodies. The proteins were visualized by western-blotting using fluorescent streptavidin. **b** U2OS-N1 cells expressing or not Tspan5, Tspan14 or Tspan15 were lysed in the presence of Brij 97 before immunoprecipitation as indicated. The composition of the immunoprecipitates was analyzed by Western blotting using a combination of biotin-labeled mAb and fluorescent streptavidin. A relative quantification of ADAM10 in the different immunoprecipitates in shown

We then analyzed by quantitative mass-spectrometry the repertoire of membrane proteins associating with ADAM10 according to the expression of Tspan5 or Tspan15 (Table 2). Surprisingly, with the exception of chondroitin sulfate proteoglycan 4 and platelet-derived growth factor receptor beta, ADAM10 did not co-immunoprecipitate the proteins specifically associated with Tspan15. In contrast, it co-immunoprecipitated a number of proteins associated with CD9 and Tspan5, including the integrin α3β1, CD9P1 and EWI-2. Importantly, these proteins were less efficiently co-immunoprecipitated with ADAM10 after Tspan15 expression than after Tspan5 expression. To confirm these findings and check that ADAM10 associated with these molecules at the cell surface, ADAM10 was immunoprecipitated after biotin labeling and Brij97 lysis. The co-immunoprecipitated proteins were eluted in RIPA buffer and identified through a second immunoprecipitation step (Fig. 8a). These data indicated that in the presence of Tspan15, ADAM10 co-immunoprecipitated a lower fraction of surface CD9, EWI-2, CD151 and integrin α3β1.

**Table 2:** Plasma membrane-associated integral proteins co-immunoprecipitated with ADAM10 from U2OS-N1 cells expressing Tspan5 or Tspan15

Protein name	Gene name	Protein abundance (area/10^6^) (number of unique peptides)			
		Tspan5		Tspan15	
Disintegrin and metalloproteinase domain-containing protein 10	ADAM10	372	(2)	662	(6)
Tetraspanin-5	TSPAN5	1370	(1)	ND	(5)
CD9 antigen	CD9	346	(1)	178	(1)
Tetraspanin-14	TSPAN14	183	(1)	203	(2)
CD81 antigen	CD81	51.2	(1)	25.9	(1)
Tetraspanin-9	TSPAN9	30.8	(1)	45.7	(1)
CD82 antigen	CD82	3.87	(1)	ND	
CD63 antigen	CD63	ND		5.96	(1)
Tetraspanin-15	TSPAN15	ND		3070	(5)
Integrin beta-1	ITGB1	684	(8)	256	(5)
Integrin alpha-3	ITGA3	984	(12)	262	(5)
Integrin alpha-6	ITGA6	132	(13)	33	(8)
Prostaglandin F2 receptor negative regulator (CD9P-1)	PTGFRN	943	(17)	383	(11)
Immunoglobulin superfamily member 8 (IgSF8) (EWI-2)	IGSF8	175	(7)	115	(5)
Choline transporter-like protein 1 (CTL-1)	SLC44A1	44.6	(2)	31.8	(3)
Lactadherin short form	MFGE8	34.7	(2)	14.1	(3)
Teneurin-3	TENM3	33.0	(3)	22.4	(1)
Adipocyte plasma membrane-associated protein	APMAP	30.9	(1)	ND	
Transferrin receptor protein 1	TFRC	23.6	(6)	38.4	(9)
Syntaxin-4	STX4	22.1	(2)	ND	
Sodium/potassium-transporting ATPase subunit alpha-1	ATP1A1	18.1	(5)	80.6	(5)
Matrix metalloproteinase-14 (MT1-MMP)	MMP14	8.64	(1)	54.0	(6)
Receptor-type tyrosine-protein phosphatase F	PTPRF	8.22	(1)	ND	
4F2 cell-surface antigen heavy chain	SLC3A2	7.66	(2)	8.44	(2)
Basal cell adhesion molecule (BCAM)	BCAM	6.95	(2)	ND	
Zinc transporter ZIP14	SLC39A14	3.37	(1)	ND	
Chondroitin sulphate proteoglycan 4	CSPG4	ND		212	(28)
Platelet-derived growth factor receptor beta	PDGFRB	ND		4.69	(1)

The table shows the integral proteins known to be present at the plasma membrane and co-immunoprecipitated with ADAM10 from cells expressing Tspan5 or Tspan15. Only proteins identified with two unique peptides are considered, except for tetraspanins and proteins identified in the Tspan5 or Tspan15 immunoprecipitates. Note that the relatively low level of ADAM10 in the samples is due to the elution of co-immunoprecipitated material in Laemmli buffer at room temperature

Finally, to validate that the reduced ability of ADAM10 to co-immunoprecipitate other cell membrane proteins in the presence of Tspan15 is not due to an impairment of the antibody to recognize a particular fraction of ADAM10, reciprocal experiments were performed. As shown in Fig. 8b, CD9, CD9P-1, CD151 and the integrin α3β1 co-immunoprecipitated a smaller fraction of ADAM10 in cells transfected with Tspan15 than in cells transfected with Tspan5 or Tspan14, or parental cells. In addition, these molecules co-immunoprecipitated Tspan5 or Tspan14, but little Tspan15.

Altogether these data indicate that Tspan15 inhibits to some extent the interaction of ADAM10 with classical constituents of the tetraspanin web.

### Differential plasma membrane localization of ADAM10 according to the expression of Tspan5 or Tspan15

The above data indicate that ADAM10 better interacts with classical components of the tetraspanin web when associated with Tspan5 than when associated with Tspan15. We then studied whether these tetraspanins differentially regulated the subcellular localization of ADAM10. Both Tspan5 and Tspan15 were expressed at the plasma membrane (as also shown in Fig. 1). Tspan15 was also enriched in an internal compartment that was identified as late endosomes as determined by its colocalization with CD63 (supplementary Figure 3). There was however no enrichment of ADAM10 in this compartment, as shown by the absence of detectable labeling of this compartment with an anti-ADAM10 mAb after permeabilization (supplementary Figure 3).

It had been previously demonstrated that several tetraspanins were enriched at the periphery of breast cancer cells [37]. Similarly, we observed an enrichment at the periphery of U2OS-N1 cells, at the plane of cell attachment, of CD9 and other molecules of the tetraspanin web (CD9P-1 or CD81). There was also an enrichment of ADAM10 at the periphery of these cells, which was reinforced when cells were incubated at 37 °C for 15 min with the anti-ADAM10 mAb 11G2 (supplementary Figure 4).

**Fig. 9:**
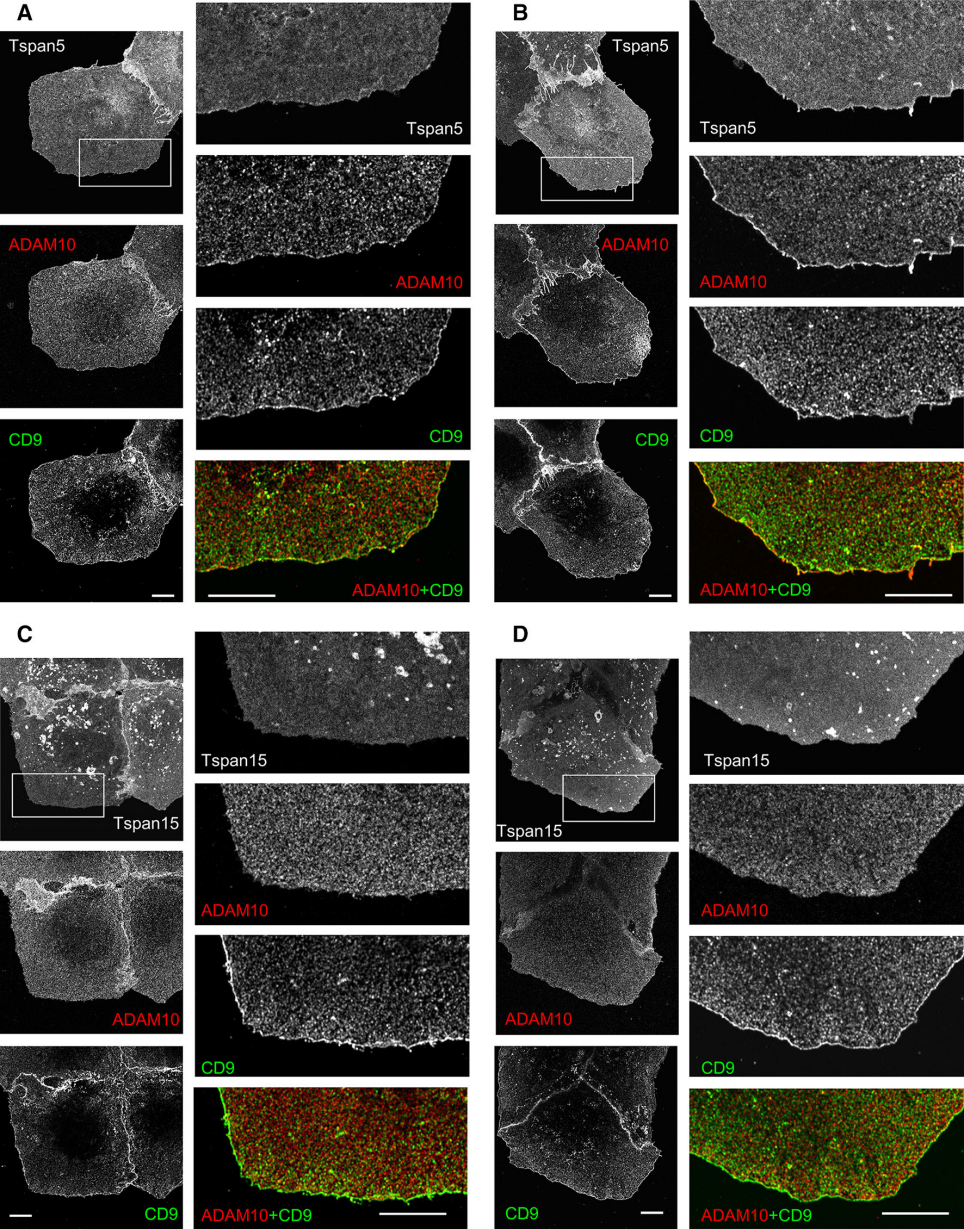
ADAM10 is enriched at the cell periphery in cell transfected with Tspan5 but not in cells transfected with Tspan15. The cells were fixed, labeled with antibodies to ADAM10 and CD9, and analyzed by confocal microscopy at the plane of cell attachment. **a** U2OS-N1/Tspan5 cells. **b** U2OS-N1/Tspan5 cells incubated for 15 min at 37 °C with the anti-ADAM10 mAb 11G2, **c** U2OS-N1/Tspan15 cells. **d** U2OS-N1/Tspan15 cells incubated for 15 min at 37 °C with the anti-ADAM10 mAb 11G2. *Bar* 10 µm

ADAM10 was also clearly enriched at the cell periphery in cells transfected with Tspan5, and as with parental U2OS-N1 cells, this peripheral labeling of ADAM10 was reinforced when cells were incubated at 37 °C for 15 min with the anti-ADAM10 mAb (Fig. 9). In contrast, there was no enrichment of ADAM10 at the cell periphery of U2OS-N1/Tspan15 cells, whether the cells were incubated with the anti-ADAM10 mAb or not.

This differential enrichment of ADAM10 at the cell periphery according to the expression of Tspan5 or Tspan15 led us to compare at the single molecule level the behavior of this protease at the cell center and at the cell periphery (supplementary Table 2). The dynamic behavior of ADAM10 in U2OS-N1/Tspan15 cells was similar at both locations. In contrast, the behavior of ADAM10 in U2OS-N1/Tspan5 cells differs according to the cell region analyzed, with Brownian trajectories being slower at the cell periphery than at the cell center.

Altogether, these data indicate that the ability of ADAM10 to localize into discrete membrane areas enriched in several tetraspanins and their partners is differentially regulated by its association with Tspan5 or Tspan15.

## Discussion

Like other surface molecules associated with the tetraspanin web, ADAM10 forms primary complexes with discrete tetraspanins. ADAM10 is, however, unusual by the fact that it associates directly with six tetraspanins, all members of a particular subgroup characterized by eight cysteines in the large extracellular domain and referred to as TspanC8 [16, 18, 19]. We now demonstrate that the TspanC8 tetraspanins have a different impact on ADAM10 dependent Notch signaling and the cleavage of several ADAM10 substrates. This is associated with a different membrane compartmentalization of ADAM10.

### TspanC8 tetraspanins have a different impact on Notch activity and the cleavage of ADAM10 substrates

The high number of tetraspanins that associate directly with ADAM10 suggests that each of these TspanC8 could confer to ADAM10 different properties. In this regard, two of the TspanC8 were shown to target ADAM10 to a late endosomal compartment in HeLa cells, whereas four of them (Tspan5, Tspan14, Tspan15 and Tspan33) increased its surface expression levels [16]. Expression of Tspan5 and Tspan14 in HeLa cells stimulated ligand-induced Notch signaling, while silencing these two tetraspanins in U2OS-N1 cells reduced Notch signaling [16]. The effect of increasing or decreasing the expression of these TspanC8 on Notch activity correlated with a change in ADAM10 expression level. It was therefore not possible to determine whether the effect on Notch signaling was a mere effect of a change of ADAM10 surface expression levels, or whether these tetraspanins also regulated the ability of ADAM10 to mediate Notch activation after ADAM10 has reached the plasma membrane. To address this question and test the effect of Tspan15 and Tspan33 on Notch activation (U2OS-N1 cells express little amounts of these 2 TspanC8), we stably expressed Tspan5, Tspan14, Tspan15 and Tspan33 in these cells. Whereas all 4 TspanC8 induced an increase in ADAM10 surface expression levels, Tspan15 and Tspan33 expression reduced by ~60 % ligand-induced Notch signaling. Expression of Tspan5 and Tspan14 did not result in an increase of Notch signaling, perhaps because this signaling is optimal in U2OS-N1 cells. The conclusion that Tspan5 and Tspan15 are, respectively, positive and negative regulators of Notch signaling is strengthened by the analysis of PC3 cells. We observed that Notch signaling in PC3 cells could be stimulated by OP9 cells expressing the Notch ligand DLL1. This stimulation was abolished by treating the cells with an ADAM10 inhibitor and a γ-secretase inhibitor, indicating the engagement of the canonical Notch pathway. We have previously shown that these cells express mainly Tspan15 and at a lower level Tspan5, as determined by RT-qPCR [16]. Importantly, silencing Tspan15 in these cells reduced ADAM10 expression level by 60 %, but increased Notch signaling twofold. In contrast, silencing Tspan5 reduced Notch signaling despite a lower impact on ADAM10 surface expression levels. Finally, we have shown that Tspan15 inhibited Notch signaling at a pre-γ-secretase step. Altogether, these data indicate that ADAM10-dependent Notch signaling is facilitated by the presence of Tspan5 (and probably Tspan14) and inhibited by the presence of Tspan15 or Tspan33. It was previously demonstrated that the *C. elegans* TspanC8 tetraspanin, Tsp-12 and the three drosophila TspanC8 were positive regulators of Notch signaling [16, 17]. The finding that Tspan15 and Tspan33 are negative regulators of Notch signaling is coherent with the fact that they are more distant to these invertebrate tetraspanins than Tspan5 and Tspan14 [16].

We have also shown that the different TspanC8 not only differentially regulate Notch activation, but also the ectodomain shedding of other ADAM10 substrates. We found that Tspan15 transfection in U2OS cells strongly reduced the formation of APP CTF in U2OS cells, whereas the transfection of Tspan5 has no effect and the transfection of Tspan14 and Tspan33 produced an intermediate effect. In PC3 cells, Tspan15 silencing slightly enhanced the generation of APP CTF, but strongly impaired the cleavage of N-cadherin, whereas Tspan5 silencing had no reproducible effect on the cleavage of these two proteins. Finally, the cleavage of CD44 was strongly reduced following Tspan5, but not Tspan15, silencing. The negative regulation by Tspan15 of APP cleavage was surprising because Prox et al. recently observed that transfection of Tspan15 increased both the expression of ADAM10 at cell surface and the cleavage of N-cadherin and APP [19]. The reasons for these discrepancies are unclear. We suggest that the effect of transfecting Tspan15 on the cleavage of various substrates might depend on the levels of other TspanC8 and of ADAM10. In cells expressing low levels of endogenous ADAM10/Tspan5 or ADAM10/Tspan14 complexes, and retention of ADAM10 in the ER as shown for HeLa cells [16], the large increase in ADAM10 expression levels produced by the expression of Tspan15 may stimulate the shedding of some of its substrates, even if ADAM10 is less active towards this particular substrate when it is associated with Tspan15 (relative to when it is associated with Tspan5 or Tspan14). In contrast, in a cell having large amount of ADAM10/Tspan5 (or Tspan14) complexes (like presumably in U2OS cells), expression of Tspan15 is likely to lead to the replacement of some of the ADAM10/Tspan5 (or Tspan14) complexes by ADAM10/Tspan15 complexes, less active towards certain substrates, and consequently a decrease in ectodomain shedding of these substrates. Further work will be necessary to validate this hypothesis.

### TspanC8 tetraspanins have a different impact on ADAM10 membrane compartmentalization

How do TspanC8 differentially affect ADAM10 activity? The simplest explanation is that Tspan5 and Tspan15 differentially regulate the interaction of ADAM10 with some of its substrates. In this regard, our mass-spectrometry analysis has shown that N-cadherin and CD44, the cleavage of which are positively regulated by Tspan15 and Tspan5 are better co-immunoprecipitated with Tspan15 and Tspan5, respectively (supplementary Table 1). Tspan5 or Tspan15 may change the ability of ADAM10 to recognize its substrates. Alternatively, they may confer to ADAM10 a different membrane compartmentalization that allow or prevent its interaction with certain substrates. Several observations are in favor of the idea that Tspan5 and Tspan15 differentially impact ADAM10 membrane compartmentalization: (1) the distribution of ADAM10 in sucrose gradients after lysis in mild detergent is different whether cells have been transfected with Tspan5 or Tspan15. (2) The ability of ADAM10 to interact with several well-characterized components of the tetraspanin web is lower after expression of Tspan15. (3) ADAM10 is not enriched together with CD9 and other components of the tetraspanin web at the periphery of cells expressing Tspan15. (4) The dynamics of ADAM10, analyzed using single molecule tracking differ whether Tspan5 or Tspan15 is transfected.

Both the transfection of Tspan5 or Tspan15 induced an increase in ADAM10 expression level. However, ADAM10 better co-immunoprecipitated classical components of the tetraspanin web after Tspan5 transfection than after Tspan15 transfection. We suggest that a better interaction with other tetraspanins explains why ADAM10 is less solubilized in the presence of Tspan5, as previously suggested for other tetraspanin/partner pairs [38]. It is also probably the reason why, in U2OS/Tspan5 cells (as compared to U2OS/Tspan15 cells), ADAM10 molecules are more confined and diffuse slower when randomly diffusing within the plasma membrane, especially at the cell periphery. Indeed, a slower diffusion may mean that ADAM10 is part of larger complexes because the diffusion of transmembrane proteins in liquid membranes is believed to decrease with the size of the diffusant [39, 40]. It may alternatively indicate more interactions with other membrane constituents [41]. Of note, the lower co-immunoprecipitation of components of the tetraspanin web with ADAM10 in U2OS/Tspan15 cells is not associated with a higher co-immunoprecipitation of other membrane proteins (Fig. 8a; Table 2), despite the fact that Tspan15 co-immunoprecipitates a number of integral proteins that are not precipitated with CD9 or Tspan5. This suggests that only the fraction of Tspan15 not associated with ADAM10 might co-immunoprecipitate these proteins. In this regard, a large fraction of Tspan15 is present in a late endocytic compartment, where it is not associated with ADAM10 as determined by the absence of detectable ADAM10 in this compartment. We therefore propose that the integral molecules co-immunoprecipitated with Tspan15, but not with Tspan5 or ADAM10, although reported to have a major cell membrane localization, represent a fraction of these molecules present in a late endocytic compartment.

Consistent with the lower interaction of ADAM10 with the tetraspanin web in the presence of Tspan15, ADAM10 lost in U2OS/Tspan15 cells its enrichment at the cell periphery (at the plane of cell attachment), where are also enriched CD9 and other CD9-associated molecules. Recent analysis of CD9 dynamics showed that CD9 cycles between tetraspanin-enriched areas (TEA) and the rest of membrane. Using Fab fragments, CD9 molecules were shown to transiently confine into these CD9-enriched areas. In contrast, when using intact mAb, CD9 molecules entering these TEA could not exit, presumably due to mAb-crosslinking of several CD9 molecules [34]. Interestingly, treatment of cells with intact anti-ADAM10 mAb resulted in the accumulation of ADAM10 molecules at the periphery of cells expressing Tspan5, but not of cells expressing Tspan15 (Figs. 9; S4). Considering all these data, we suggest that the cell periphery can be assimilated to TEA, and that the local enrichment of CD9, ADAM10 and other molecules is a consequence of simultaneous confinement of several of these molecules.

Previous studies have demonstrated that a tetraspanin can regulate the membrane environment of their partner proteins (defined as a molecule interacting directly with this tetraspanin), as determined using sucrose gradient fractionation after cell lysis [38, 42], and regulate the interaction of this partner to other tetraspanins [38, 43, 44]. More recently, several studies have shown that a tetraspanin can change the dynamics of its partner protein [45, 46]. In this study, using a combination of several approaches including single molecule tracking and quantitative mass-spectrometry analysis of tetraspanins and ADAM10 complexes, we highlight a unique example in which two different tetraspanins have a different impact on the function of their partner protein through the regulation of its membrane compartmentalization. Further work will be necessary to determine precisely the mechanisms responsible for the different compartmentalization of the various ADAM10/TspanC8 complexes.

## Materials and methods

### Antibodies, plasmids, siRNAs and inhibitors

The rabbit polyclonal anti-Notch1 antibody, as well as the mAb directed to human ADAM10 (11G2, IgG1), CD9 (TS9, IgG1 and TS9b, IgG2b), CD81 (TS81, IgG2a), CD63 (TS63, IgG1), CD151 (TS151, IgG1), CD9P-1 (1F11, IgG1) and EWI-2 (8A12, IgG2a) have been previously described [13, 25, 28, 47]. The CD151 mAb 11B1G4 [48] and the rabbit polyclonal antibodies to the cytoplasmic domain of the integrin α3 [49], APP, and CD44 were provided by L. Ashman, A Sonnenberg, W. Annaert and S. Manié, respectively. The rabbit anti-α2 integrin antibody, the anti-α3 integrin mAb Mkid2 and the anti-CD97 mAb were purchased from Millipore. The mouse mAb anti-IgSF3 was from R&D systems and the anti-transferrin receptor mAb (H68.4) was from Life technologies. The rabbit polyclonal anti-GFP antibody was from Santa Cruz. All plasmids used in this study were previously described [16].

siRNAs were obtained from Invitrogen (Stealth) or Eurogentec. All silenced by >90 % their target at the RNA level in PC3 cells as determined by RT-QPCR, and by >80 % at the protein level as determined by flow-cytometry analysis using U2OS cells transfected with the respective GFP-tagged target.

- siTspan15 #1 (Stealth): ACAACCUGUACCUUCUCCAAGCAUU.
- siTspan15 #2 (Stealth): GGAUCUGCCTCAUCAUGGAGCUCAU.
- siTspan15 #3 (Stealth): GAGGACTACCGAGATTGGAGCAAGA
- SiTspan5 #1 (Stealth): AUGUCAUCCCGAUAUGCUCUGAUGU
- siTspan5 #2: GCUGAUGAUUGGAACCUAA-dTdT
- siTspan5 #3: GACCAGCUGUAUUUCUUUA-dTdT
- siTspan5 #4: GAGCAUAUCGGGAUGACAU-dTdT

Control siRNA: Stealth RNAi Negative Control Medium GC and UUUGUAAUCGUCGAUACCC-dTdT

DAPT and GI254023X were purchased from Merck Millipore and Sigma Aldrich, respectively.

### Cell culture and generation of cells expressing GFP-tagged tetraspanins

OP9 cells expressing the human Notch ligand DLL-1 (OP9- DLL-1) and the human osteosarcoma cell line U2OS expressing human Notch1 (U2OS-N1) have been previously described [47, 50]. U2OS-N1 cells stably expressing GFP-tagged tetraspanins were obtained by transfection using either Fugene 6 (Promega) or Jetprime (Polyplus transfection,) and cell sorting using a FACS Aria cell sorter (Beckton–Dickinson). Although we sorted cells with the highest level of expression, a fraction of cells progressively lost the expression of the transfected tetraspanin, possibly due to the silencing of the CMV promoter [51]. The expression of GFP-tagged TspanC8 was routinely checked to use only cell populations with sufficient expression of the transfected tetraspanin (Fig. 1a)

U2OS-N1 and PC3 (a prostate carcinoma cell line) cells were cultured in DMEM and OP9 cells were cultured in alphaMEM. Both media were supplemented with 10 % FCS and antibiotics.

### Flow-cytometry analysis

Cell were detached with trypsin, washed twice in complete DMEM and incubated for 30 min at 4 °C with 10 µg/ml primary antibody. After three washings, the cells were incubated for 30 min at 4 °C with a Phycoerythrin-conjugated F(ab’)_2_ goat anti-mouse antibody The cells were analyzed using an Accuri C6 flow-cytometer (Becton–Dickinson), using appropriate compensations.

### Analysis of Notch activity

This analysis was performed as previously described (Moretti et al. 2010): U2OS-N1 or PC3 cells were seeded at the concentration of 25,000 cells/cm^2^. Silencing was performed at this step using Interferin (PolyPlus transfection) and 10 nM siRNA according to the manufacturer’s reverse procedure. Cells were transfected 24 h later with the CSL reporter and Renilla plasmids using FuGene6 (Promega). 24 h later, cells were co-cultured with OP9 or OP9-DLL1 at 35,000 cells/cm^2^. The activities of firefly and Renilla luciferases were determined using a Dual luciferase reporter assay (Promega) according to the manufacturer’s instructions. To study the effect of immobilized recombinant DLL1 on Notch activation, cells transfected with the CSL reporter and Renilla plasmids were culture for 20–24 h in plates previously coated with a rabbit anti-human Fc polyclonal antibody (Jackson), and with the conditioned medium of HEK293 cells expressing DLL1 (provided by Dr. Weinmaster [52]). Statistical analysis was performed using one-way Anova followed by the Tukey multiple comparison test.

### Biotin labeling of surface proteins and immunoprecipitation

Biotin labeling of surface proteins and immunoprecipitations were performed as previously described [13, 28]. Briefly, cells were lysed in a lysis buffer (30 mM Tris pH 7.4, 150 mM NaCl, 1 mM CaCl_2_, 1 mM MgCl_2_, protease inhibitors) supplemented with 1 % Brij 97. After 30 min incubation at 4 °C, the insoluble material was removed by centrifugation at 10,000×*g* and the cell lysate was precleared by addition of heat inactivated goat serum and protein G Sepharose beads (GE Healthcare). Proteins were then immunoprecipitated by adding 1 µg mAb and 10 µl protein G-Sepharose beads to 200–400 µl of the lysate or using GFP-trap beads (Chromotek). The immunoprecipitated proteins were separated by SDS-polyacrylamide gel electrophoresis and transferred to a PVDF membrane (Amersham). Western blotting on GFP-trap immunoprecipitates was performed using appropriate combinations of primary and fluorescent secondary antibodies. Western blotting on immmunoprecipitations performed with mouse mAbs was performed using biotin-labeled antibodies and fluorescent streptavidin. All acquisitions were performed using the Odyssey Infrared Imaging System (LI-COR Biosciences).

### Analysis of protein cleavage

The cells treated or not with siRNA were incubated or not for 24 h with DMSO, DAPT or a combination of DAPT and GI254023X (both at 3 µM). They were lysed directly in Laemli buffer for Western-blot analysis. For each protein analyzed, the intensity of the band corresponding to the CTF was measured using the Odyssey Software, and normalized to the amount of the intact proteins. Results are expressed as a percent of CTF production observed in the control sample.

### Equilibrium density gradient centrifugation

The cells were pelleted and lysed in ice-cold lysis buffer supplemented with 2 % Brij 98. After a 30 min incubation on ice, the preparation was made 40 % with respect to sucrose, in the lysis buffer without detergent. Then, 0.8 ml of lysate–sucrose mixture was sequentially overlaid with 2 ml of 30 % sucrose and 1 ml of 4 % sucrose prepared in the same buffer, without detergent, and the mixture was centrifuged at 200,000*g* for 14–16 h in a SW50.1 rotor (Beckman). The gradient was fractionated in 0.5-ml fractions from the top of the tube and analyzed by Western blot using appropriate antibodies.

### Single molecule tracking experiments

Single molecule tracking experiments were carried out as previously described [34, 36]. Briefly, cells were incubated in DMEM at 37 °C for 10 min with Atto647N-labeled Fab fragments of the anti-ADAM10 mAb. Images were acquired using a homemade objective-type TIRF set-up equipped with a Plan Fluor 100×/1.45 NA objective (Zeiss), with a 100 ms integration time. All the movies were analyzed using a homemade software (named ‘PaTrack’) implemented in visual C++. Trajectories were constructed using the individual diffraction limited signal of each molecule. The centre of each fluorescence peak was determined with subpixel resolution by fitting a two-dimensional elliptical Gaussian function. The two-dimensional trajectories of single molecules were constructed frame per frame. Only trajectories containing at least 40 points were retained. Diffusion coefficient values were determined from a linear fit to the MSD (mean square displacement)-t plots between the first and the fourth points (D1–4) according to the equation MSD(t) = 4Dt. The determination of the motional modes was performed using a homemade algorithm based on a neural network that has been trained using synthetic trajectories to detect pure Brownian, confined and directed motion modes (Dosset et al. submitted). Due to a sliding window, the trajectory is analyzed and the different modes detected within a trajectory for segments larger than 10 frames. Once the motion mode is identified, the different segments are analyzed by plotting the MSD versus time lag. The MSD curve is linearly fitted (Brownian) or adjusted with a quadratic curve (4Dt + *v*_2_*t*_2_) (directed diffusion) or exponential curve L2/3[1 − exp(−12Dt/L2)] (confined diffusion), where L is the side of a square domain, the confinement diameter being related to *L* by dconf = (2/√*L*). The algorithm has been tested with simulated trajectories displaying pure Brownian, confined or directed behaviour or a combination of these three modes and successfully applied to a set of single-molecule experiments previously recorded for tetraspanins diffusing into plasma membrane [34, 35]. Statistical analysis was performed using the Mann–Whitney *U* test.

### Immunostainings and confocal microscopy

The cells grown in complete medium for 48 h on coverslips were incubated or not with the anti-ADAM10 mAb 11G2 for 15 min at 37 °C and fixed for 15 min with 4 % paraformaldehyde at room temperature. After three washing and blocking for 20 min with 50 mM NH_4_Cl in PBS, the cells were then incubated for 30 min with 10–20 µg/ml of antibodies in PBS supplemented with 0.1 % BSA at room temperature. Double labeling was performed using antibodies of different subclasses, revealed with Alexa Fluor 568 and Alexa Fluor 647-labeled goat anti-mouse IgG subclasses. The cells were mounted in Mowiol 4-88 supplemented with DABCO (Sigma) and DAPI and examined with a Leica SP5 confocal microscope (63× objective, 1.4 numerical aperture, zoom 3 and 6)

### Identification of tetraspanin and ADAM10-associated proteins by mass-spectrometry

Cells expressing GFP-tagged CD9, Tspan5 or Tspan15 were lysed in lysis buffer supplemented with 1 % Brij97 and protease inhibitors. The insoluble material was removed by centrifugation at 12,000*g* for 15 min, and the tetraspanins were immunoprecipitated using GFP-trap beads. To identify and exclude from further analysis the proteins that are not specifically co-immunoprecipitated with the target tetraspanins, non-transfected cells and cells transfected with GFP were similarly processed. Endogenous CD9 and ADAM10 proteins were immunoprecipitated using Sepharose 4B beads coupled to the anti-CD9 mAb TS9 or the anti-ADAM10 mAb 11G2, respectively. The non-specifically bound proteins were identified by performing a similar experiment using an irrelevant mAb of the same subclass.

The proteins were separated by electrophoresis using 4–12 % Tris-bis polyacrylamide gel (nupage, Invitrogen) under reducing conditions and stained with colloidal Coomassie Blue (imperial stain, Pierce). Gels slices containing proteins were excised and destained in 200 µl of 0.1 M NH_4_HCO_3_/acetonitrile v/v for 15 min, centrifuged and swollen in H_2_O repeatedly until complete destaining. Gel pieces were then incubated in 150 µl of 100 % acetonitrile for 10 min and dried. This was followed by rehydration in 0.1 M NH_4_HCO_3_ containing 30 mg/ml TCEP (Tris(2-carboxyethyl)phosphine hydrochloride) for 10 min at room temperature. The TCEP solution was replaced with 55 mM iodoacetamide in 0.1 M NH_4_HCO_3_ for 30 min at room temperature in the dark. The gel pieces were washed in 150 µl of 0.1 M NH_4_HCO_3_ for 10 min, before addition of 150 µl acetonitrile for 15 min, and then dehydrated in 100 % acetonitrile, and dried. The gel pieces were then covered with a solution of trypsin (13.33 µg/ml in 0.1 M NH_4_HCO_3_) and incubated overnight at 37 °C. After supernatant retrieval, the gel fragments were extracted twice by addition of 20 µl of acetonitrile/5 % formic acid (70/30 v/v) and incubation for 20 min at 37 °C. Supernatants were pooled, dried and rehydrated in acetonitrile/formic acid/H_2_O (3 %/0.5 %/96.5 % v/v).

LC–MS/MS analyses were performed using an ESI linear ion trap-Orbitrap hybrid mass spectrometer (LTQ-Orbitrap Velos, Thermo Fisher Scientific, Bremen, Germany) coupled on line with a nano-HPLC system (Ultimate 3000; *Dionex*) for liquid chromatography. 5 µl peptide solution was injected in the system using a pre-concentration column (C18 trap column—PepMap C18, 300 μmID × 5 mm, 5 μm particle size and 100 Å pore size; *Dionex*). The nano-column used in this study was a PepMap C18 reverse phase (Acclaim pepmap RSLC 75 µm × 15 cm, nanoViper C18, 2 µm, 100 Å). A linear 45 min gradient (flow rate, 300 nl/min) from 4 to 55 % acetonitrile in 0.1 % (v/v) was applied. After the acquisition of a full MS scan by the Orbitrap at high resolution (30000 resolution, *m*/*z* range were 380–1700 Da) in the first scan event, the five most intense ions present were subsequently isolated for fragmentation (MS/MS scan). The collision energy for the MS/MS scan events was pre-set at a value of 35 %, the isolation window was set at 3 Da, Dynamic exclusion option was enabled. The capillary voltage was set at 1.6 kV and the capillary temperature was 275 °C.

The data were analyzed using the Proteome Discoverer 1.4 software. The MS/MS spectra were searched against the Uniprot human Protein Database. The maximal allowed mass tolerance was set to 10 ppm for precursor ions and to 0.6 Da for fragment ions. Peptides mass is searched between 350 and 5000 Da with time retention from 10 to 50 min. Enzyme specificity was set to trypsin with a maximum of one missed cleavage. Carbamidomethylation of cysteine was set as a fixed modification. Protein N-terminal acetylation, oxidation of methionine, and carbamidomethylation of histidine, aspartic acid and glutamic acid were selected as variable modifications. Peptide identifications were validated by determination of false positives by Target decoy PSM validator. It is high if the false positive rate (FDR or false Discovery rate) is less than 1 %, low if the FDR is greater than 5 % and average (medium between 1 and 5 %). Peptide identification Xcorr was calculated by the correlation of MS/MS experimental spectrum compared with the theoretical MS/MS spectrum generated by the Proteome Discoverer 1.4 software. A relative quantitation was performed with the Proteome Discoverer-integrated label-free method which consists in comparing the mean peaks area of the three best peptides of a given protein. The method of calculation is three dimensional relying on retention time, ion intensity and *m/z* ratio of the peptide, with a mass error lower than 2 ppm. Proteins were considered only if they were identified with more than two peptides corresponding to only one protein (unique peptides), except for tetraspanins.

## Electronic supplementary material

Below is the link to the electronic supplementary material.

Supplementary Table 1: full list of plasma membrane associated integral proteins co-immunoprecipitated with CD9, Tspan5 or Tspan15 The table show the integral proteins known to be present at the plasma membrane and co-immunoprecipitated with a CD9 mAb from U2OS-N1 cells, or with GFP-tagged CD9, Tspan5 or Tspan15 using GFP trap beads, from cells transfected with the respective tetraspanin. The subcellular location is provided by uniprot. The other columns correspond to the results of protein identification performed using proteome discoverer: score, number of unique peptides, area (relative abundance), total number of peptides corresponding to the protein, number of PSM (peptide-spectrum match). Supplementary Table 2: Comparison of ADAM10 ADC and distribution of the different modes of diffusion at the cell periphery or center (XLSX 36 kb)

Supplementary material 2 (PDF 2418 kb)

Movies: U2OS-N1 cells, as well as cells transfected with Tspan5 and Tspan15 (respectively movies 1, 2 and 3) were incubated with Atto647-labeled anti-ADAM10 antibody. Images were acquired by time-lapse TIRF microscopy using a Zeiss AxioOberver A1 equipped with a 100x/1.45NA objective. Frames were taken every 100 ms. The movies are played in real time (AVI 2060 kb)

Supplementary material 4 (AVI 1951 kb)

Supplementary material 5 (AVI 1167 kb)
