# Supplementary material for: TspanC8 tetraspanins differentially regulate the cleavage of ADAM10 substrates, Notch activation and ADAM10 membrane compartmentalization
Source: Cell Mol Life Sci. 2015 Dec 19;73(9):1895–915. doi: 10.1007/s00018-015-2111-z (PMC4819958; doi:10.1007/s00018-015-2111-z)
Supplement: Supplementary file 2 — Supplementary material 2 (PDF 2418 kb) [file 18_2015_2111_MOESM2_ESM.pdf]

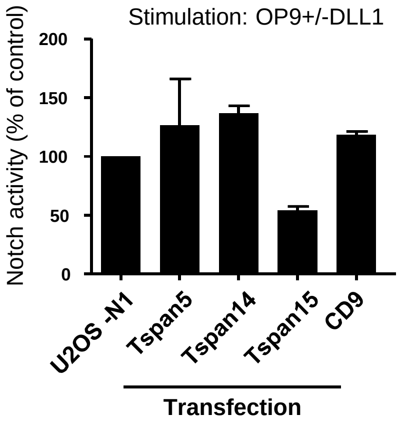

Fig. S1: Activation of Notch signaling in an independent series of U2OS-N1 cells transfected with the various TspanC8 or CD9

Notch activity of U2OS-N1 cells stably expressing or not the indicated tetraspanins, measured using a CSL reporter luciferase assay. Notch was activated by incubating the cells with OP9-DLL1 cells for 20-24h. The figure shows the mean+/-SEM of 3 independent experiments in duplicate. In each experiment, the signal obtained is expressed as a percentage of the signal observed for non-transfected U2OS cells.

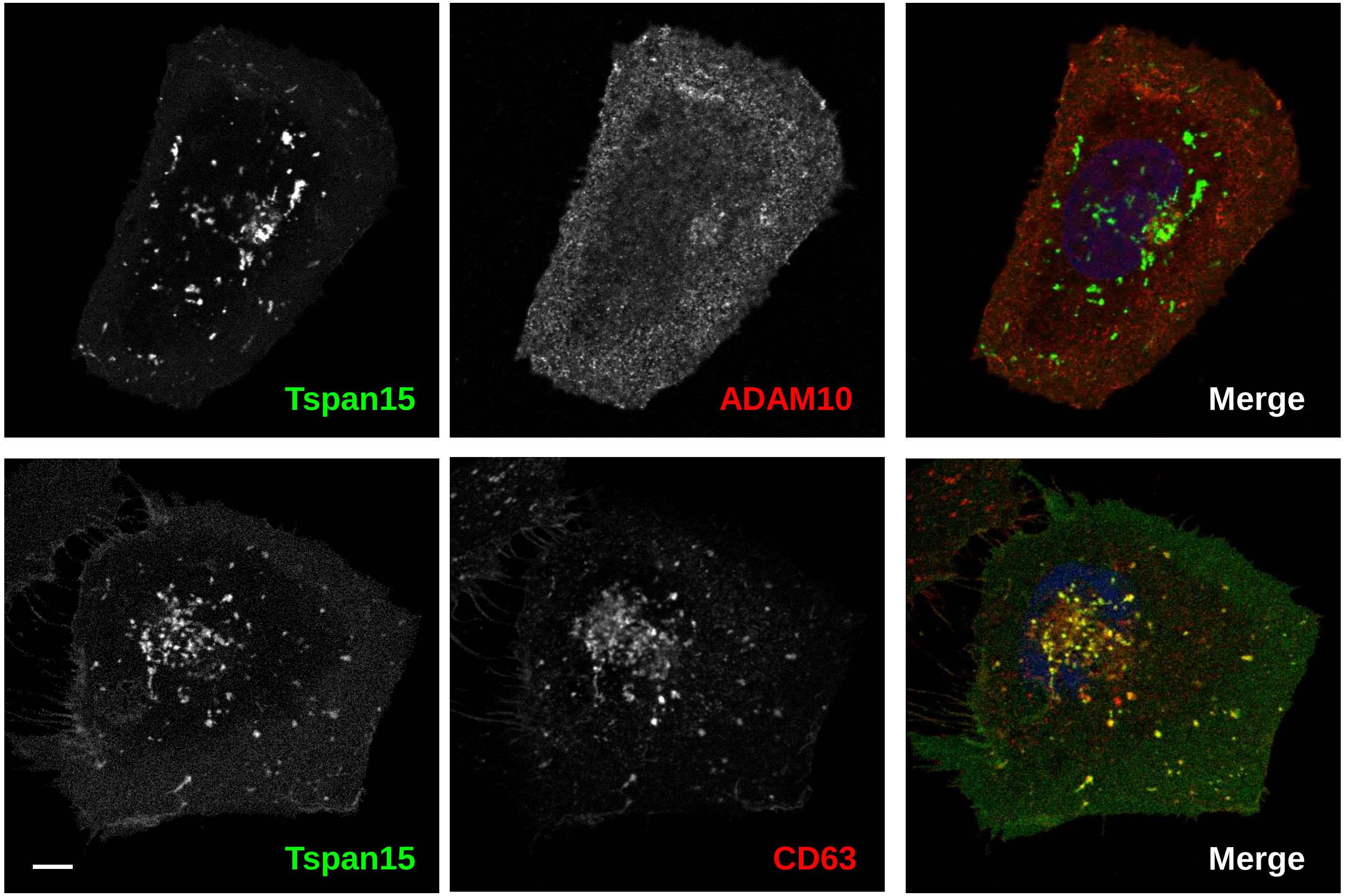

Fig. S2: Tspan15 but not ADAM10, is enriched in a late endocytic compartment
U2OS-N1/tspan15 cells were fixed, permeabilized with saponin, labelled with antibodies to CD63 or ADAM10, and analyzed by confocal microscopy. Bar: 10μm.

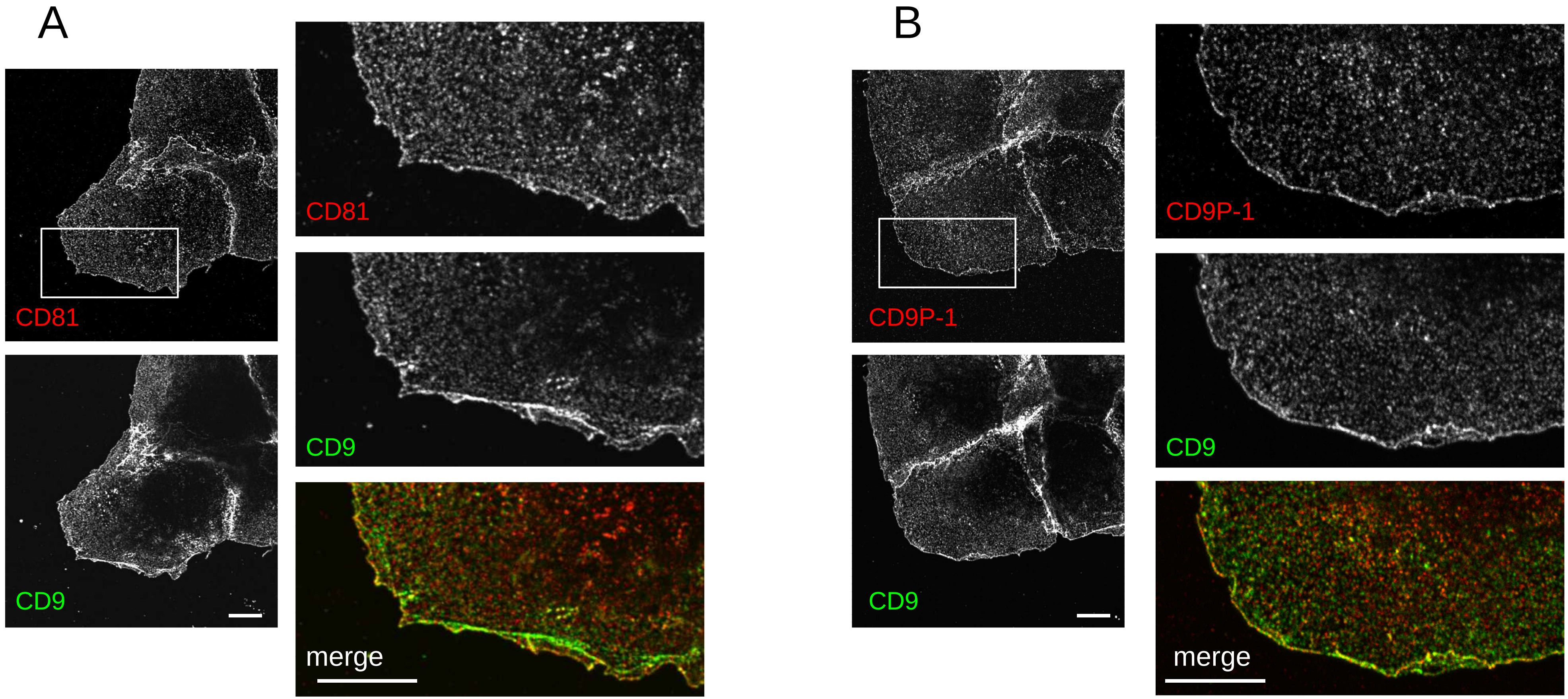

Fig. S3: ADAM10, CD81 and CD9P-1 are enriched at the cell periphery in U2OS cells
The cells were fixed, labelled with antibodies to CD9 and either ADAM10, CD9P-1 or CD81, and analyzed by confocal microscopy at the plane of cell attachment.
(A) CD81; (B): CD9P-1; Bar: 10µm.

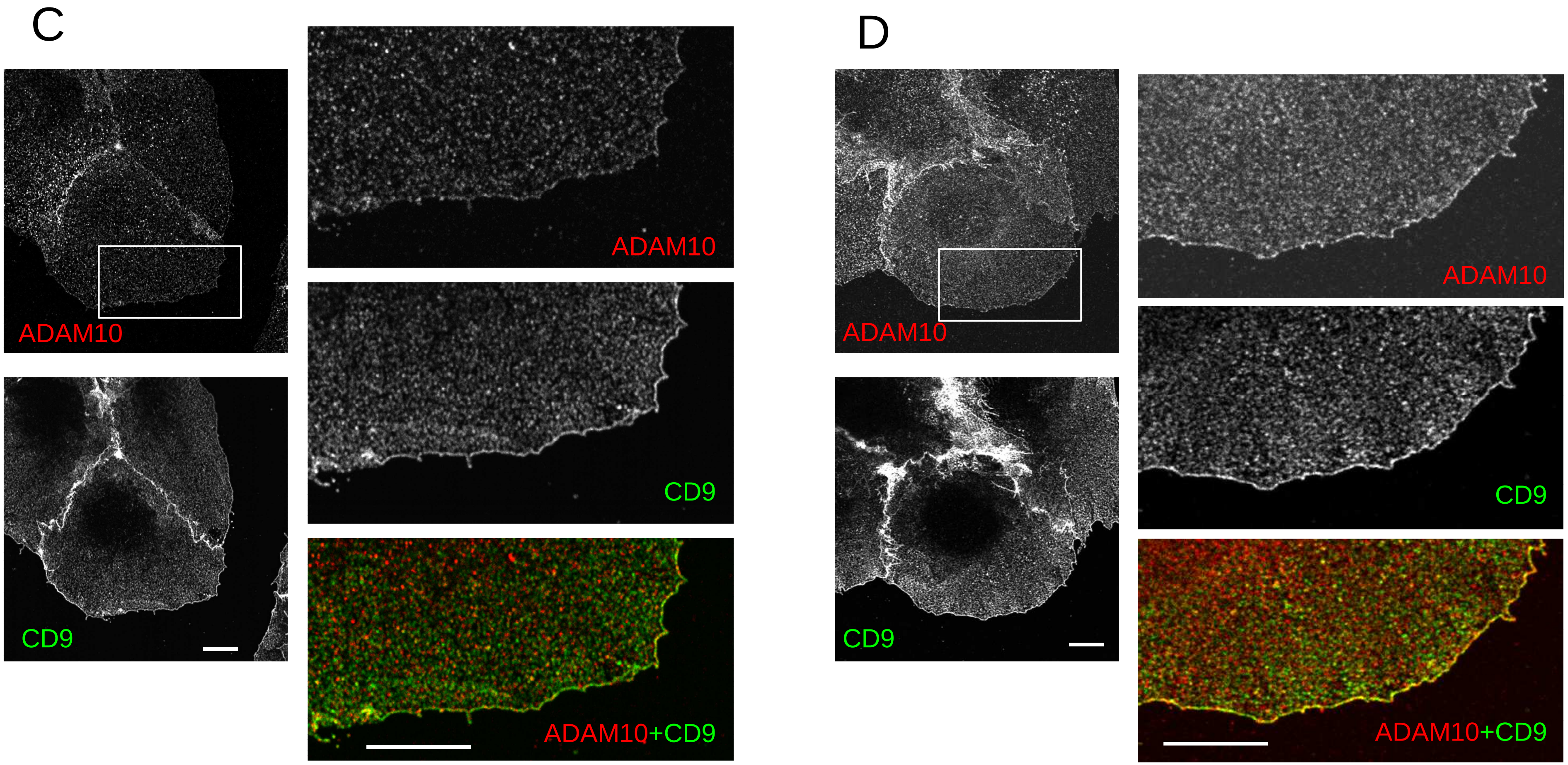

Fig. S3 (continued): ADAM10, CD81 and CD9P-1 are enriched at the cell periphery in U2OS cells
The cells were fixed, labelled with antibodies to CD9 and either ADAM10, CD9P-1 or CD81, and analyzed by confocal microscopy at the plane of cell attachment.
(C) ADAM10; (D): ADAM10 in cells incubated for 15 min with the anti ADAM10 mAb 11G2.
Bar: 10µm.

ADAM10 Apparent Diffusion Coefficients ± SEM (µm²/s)/ (% of diffusion modes)				
		Cell type		
**Trajectories**		**U2OS-N1**	**U2OS/Tspan5**	**U2OS/Tspan15**
**All**	Periphery	0,063 ± 0,026	0,062 ± 0,024	0,105 ± 0,031
	Center	0,075 ± 0,030	0,079 ± 0,030	0,103 ± 0,032
**Brownians**	Periphery	0,103 ± 0,027 (54)	0,098 ± 0,026 (54)	0,137 ± 0,030 (69)
	Center	0,124 ± 0,032 (53)	0,123 ± 0,028 (58)	0,142 ± 0,030 (68)
**Confined**	Periphery	0,007 ± 0,004 (25)	0,010 ± 0,006 (23)	0,009 ± 0,006 (16)
	Center	0,007 ± 0,003 (27)	0,008 ± 0,005 (24)	0,009 ± 0,007 (17)
**Mixed**	Periphery	0,029 ± 0,012 (21)	0,028 ± 0,009 (23)	0,045 ± 0,017 (15)
	Center	0,031 ± 0,012 (20)	0,032 ± 0,013 (18)	0,035 ± 0,014 (15)

Supplementary Table 2 : Comparison of ADAM10 ADC and distribution of the different modes of diffusion at the cell periphery or center
